# Fabrication and Characterization of the Core-Shell Structure of Poly(3-Hydroxybutyrate-4-Hydroxybutyrate) Nanofiber Scaffolds

**DOI:** 10.1155/2021/8868431

**Published:** 2021-01-28

**Authors:** Wentai Guo, Zifeng Yang, Xiusen Qin, Yingqi Wei, Chuangkun Li, Rongkang Huang, Chen Zhou, Huaiming Wang, Lin Jin, Hui Wang

**Affiliations:** ^1^Department of Colorectal Surgery, The Sixth Affiliated Hospital of Sun Yat-sen University, Guangzhou 510655, China; ^2^Guangdong Provincial Key Laboratory of Colorectal and Pelvic Floor Diseases, The Sixth Affiliated Hospital of Sun Yat-sen University, Guangzhou 510655, China; ^3^Guanghua School of Stomatology, Hospital of Stomatology, and Guangdong Provincial Key Laboratory of Stomatology, Sun Yat-sen University, Guangzhou 510030, China; ^4^The Fifth Affiliated Hospital of Guangzhou Medical University, Guangzhou 510700, China

## Abstract

Tissue engineering scaffolds with nanofibrous structures provide positive support for cell proliferation and differentiation in biomedical fields. These scaffolds are widely used for defective tissue repair and drug delivery. However, the degradation performance and mechanical properties of scaffolds are often unsatisfactory. Here, we successfully prepared a novel poly(3-hydroxybutyrate-4-hydroxybutyrate)/polypyrrole (P34HB-PPy) core-shell nanofiber structure scaffold with electrospinning and in situ surface polymerization technology. The obtained composite scaffold showed good mechanical properties, hydrophilicity, and thermal stability based on the universal material testing machine, contact angle measuring system, thermogravimetric analyzer, and other methods. The results of the in vitro bone marrow-derived mesenchymal stem cells (BMSCs) culture showed that the P34HB-PPy composite scaffold effectively mimicked the extracellular matrix (ECM) and exhibited good cell retention and proliferative capacity. More importantly, P34HB is a controllable degradable polyester material, and its degradation product 3-hydroxybutyric acid (3-HB) is an energy metabolite that can promote cell growth and proliferation. These results strongly support the application potential of P34HB-PPy composite scaffolds in biomedical fields, such as tissue engineering and soft tissue repair.

## 1. Introduction

Tissue engineering scaffolds with nanofibrous structures can provide skeletal support for cell proliferation and differentiation in biomedical fields [1–3]. These structures can be combined with tissue cells and implanted in vivo to replace and repair defective tissue. At present, such scaffolds have been studied in bone [4, 5], cartilage [6, 7], blood vessels [8, 9], and nerves [10, 11]. Applications include the fields of drug delivery [12, 13], biosensors [14, 15], and tissue engineering repair. The ideal tissue engineering scaffold exhibits good biocompatibility, surface activity, degradability, and mechanical strength as well as appropriate pore size and porosity. Therefore, the manufacturing process is vital and directly determines the initial performance of the scaffold. Additive manufacturing (AM) technology has made great contributions to achieving this goal [16]. However, the manufacturing of fiber scaffolds has many limitations, such as the need for special manufacturing technology, and the low resolution of the scaffold leads to the adhesion of fiber structures to each other, which is not conducive to cell adhesion and proliferation [17].

Nanofiber scaffolds are representative of tissue engineering scaffolds. Scaffolds have a highly porous fiber structure that mimics the natural extracellular matrix (ECM), provides a microenvironment for cell growth, and plays a critical role in maintaining tissue structure and mechanical properties while guiding tissue regeneration [18–20]. Various manufacturing technologies for nanofiber scaffolds have been developed, such as thermally induced phase separation [21], solvent casting [22], gas foaming [23], and emulsion freeze-drying [24]. Additionally, electrospinning is a commonly used manufacturing process that can be used to manufacture nanofibers with different diameters and different directions [25, 26]. In this technique, a polymer solution is spray-spun under a strong electric field to obtain nanofiber filaments. However, the materials used for electrospinning fiber scaffolds are limited, such as polylactic acid (PLA) [27], polycaprolactone (PCL) [28], and other synthetic polymers, which have poor degradation performance and can be easily damaged by external forces. To date, there have been multiple attempts to overcome these shortcomings, such as the incorporation of graphene nanoflakes, SiO_2_, and other nanomaterials to improve the mechanical properties of fiber scaffolds [29, 30]. However, these materials still have issues such as poor biocompatibility and uncontrollable degradation.

Polyhydroxyalkanoates (PHA) are linear saturated polyesters produced by microbial fermentation. The degradation cycle is expected to be 9-12 months with controllable degradation, biocompatibility, and thermal stability. The structures of PHAs are diverse, and different material properties can be obtained according to the different arrangements and chain lengths of the monomers [31, 32]. Among them, poly(3-hydroxybutyrate-4-hydroxybutyrate) (P34HB) is the latest generation of biodegradable PHA materials. To date, research on P34HB has focused on the nervous system, bone, and cartilage [33, 34]. The good mechanical properties of P34HB indicate that it has great application potential as a tissue engineering scaffold.

In addition to the effect of the substrate, surface modification also plays a vital role in practical applications [35]. For example, composite scaffolds can have an antiadhesion function by coating polydopamine material [36] or an antibacterial ability by adding nanosilver particles [37]. In previous studies, we reported that 3D fluffy cell growth scaffolds made of the conductive polymer polypyrrole (PPy) show excellent biocompatibility and controllable chemical modification [38]. Several PPy microstructural scaffolds have been reported, including microcarriers, hydrogels, and fibrous networks [39, 40]. Nevertheless, the poor mechanical properties of PPy scaffolds make necessitate their combination with substrate materials to improve performance, which limits the application of PPy in the field of tissue engineering scaffolds. Therefore, it is necessary to develop a new type of simple and high-strength composite scaffold based on PPy to meet the complex requirements of biomedical applications.

In this study, we report a novel approach to prepare composite nanofiber scaffolds with suitable mechanical properties for cell growth. First, P34HB fiber scaffolds were prepared by using solution-assisted electrospinning technology. Then, the surface of the P34HB fiber scaffold was coated with PPy using an in situ polymerization process to achieve a core-shell fiber structure composite scaffold. Compared with the previous single-layer network scaffolds, P34HB in a core-shell fiber structure can provide durable and stable mechanical strength and functional group support, whereas PPy on the surface can better imitate an ECM and improve the overall performance of the scaffolds. The morphology and mechanical properties of the P34HB-PPy composite scaffolds were further studied, and the effect of composite scaffolds on the behavior of bone marrow-derived human mesenchymal stem cells (BMSCs) was also studied. The results revealed the potential application of P34HB-PPy composite scaffolds in biomedical fields, such as skin wound repair, meningeal defect repair, and peritoneal defect repair.

## 2. Experimental Section

### 2.1. Materials

P34HB (M.W. 800 kDa) was purchased from Medpha Technology Co., Ltd. (Zhuhai, China). Pyrrole (98%) and iron (III) chloride (98%) were acquired from Sigma-Aldrich. All other chemicals were purchased from Sigma-Aldrich without special instructions and were used as is without further purification.

### 2.2. Fabrication of the Core-Shell Structure of the P34HB-PPy Scaffold

The P34HB fiber scaffold was prepared using an improved electrospinning process. First, high-purity P34HB copolymer powder was dissolved in dichloromethane/*N*,*N*-dimethyl-formamide (DCM/DMF, v/v = 8/2) to prepare a mixed solution with a concentration of 100 mg/mL and was stirred uniformly until the solution became clear and viscous. The mixed solution was added to a syringe equipped with a 0.22 blunt needle tip, and the needle nozzle was fixed and advanced by a syringe pump at a rate of 1.5 mL/h. At room temperature, a voltage of 10-20 kV was supplied by a high DC power supply between the tip of the needle and the collector at 12-18 cm. Subsequently, the nanofibers were collected by ethanol solution. The collected nanofibers were washed 3-5 times with deionized water, freeze-dried for 12 h, and finally pressed into a film to obtain a P34HB fiber scaffold membrane.

The preparation of the P34HB-PPy core-shell structure scaffold proceeded as follows. PPy was coated on the P34HB scaffold by the in situ surface polymerization of pyrrole with FeCl_3_ as an oxidant. Briefly, 1.36 g of FeCl_3_ was added to 100 mL of deionized water and was well stirred. Then, 0.244 g of pyrrole was dissolved in an equal volume of deionized water, and the two aqueous solutions were mixed uniformly. At room temperature, the P34HB fiber scaffold membrane was immersed in the above-mixed solution, ultrasonically shaken (50 kHz, output power 400 W) for 1 h, washed with deionized water 3-5 times, and dried under vacuum to obtain a core-shell structure of the P34HB-PPy fiber scaffold membrane.

### 2.3. Characterization of the Scaffold

#### 2.3.1. Scanning Electron Microscopy (SEM)

The morphology of the P34HB and P34HB-PPy fiber scaffolds was obtained by field emission scanning electron microscopy (SEM, Hitachi S-4800, Japan) at an acceleration voltage of 20 kV. The ImageJ software was applied to evaluate the average diameters and the distribution of fibers by measuring the diameters of 100 fibers in a randomly selected field from the SEM images.

#### 2.3.2. Fourier Transformer Infrared Spectroscopy (FT-IR) Analysis

The surface chemistry of the scaffolds was analyzed at room temperature on an FT-IR spectrometer (TENSOR 27, BRUKER, Germany).

#### 2.3.3. Thermogravimetric Analysis (TGA)

The thermal stability of the scaffolds was investigated in a thermogravimetric analyzer (TGA, Netzsch TG-209, Germany). The temperature ranged from 0 to 800°C and was heated at a rate of 10°C/min under a nitrogen atmosphere (100 mL/min).

#### 2.3.4. Water Contact Angle

The static contact angle of the water droplets on the scaffolds was determined with a contact angle measuring system (JGA-360A, China). Four different points of a droplet on each sample were measured, and images were captured with a charge-coupled device camera (KGV-5000, Japan). Three different readings were recorded for each point.

#### 2.3.5. Tensile Testing

The mechanical strength of the scaffolds was evaluated with a universal material testing machine (WD-5A, Guangzhou Experimental Instrument Factory, China). The preload was 0.01 N, and the crosshead speed was set at 10 mm/min. A 10 × 10 mm piece of the scaffold was held on the surface by clips and placed into the tensile machine at room temperature. Stress-strain curves and parameters were also obtained.

#### 2.3.6. Degradation Test

An enzymatic degradation experiment was performed in a Petri dish containing a phosphate buffer saline(PBS, pH=7.2) and an enzyme solution of lipase (Macklin, China) [41, 42]. The P34HB and P34HB-PPy fiber scaffold membranes (10 × 10 mm^2^) were placed in a petri dish and maintained at 37°C in a constant temperature shaker with shaking at 120 rpm. During the reaction, samples were obtained regularly every day for 7 days, washed with distilled water, and dried to constant weight in a vacuum oven. Finally, the biodegradation rate was calculated based on the mass loss before and after sample degradation.

### 2.4. Cell Culture

Bone marrow-derived mesenchymal stem cells (BMSCs, SCSP-405) were purchased from the Cell Bank of the Chinese Academy of Sciences. The cells were resuspended in a complete culture medium consisting of mesenchymal stem cell medium (MSCM) supplemented with 5% w/v fetal bovine serum (FBS), 0.5 mL mesenchymal stem cell growth additive, and 0.5 mL penicillin/streptomycin solution and cultured in a carbon dioxide cell incubator (37°C, 5% CO_2_). The complete medium was replaced every two days to achieve cell proliferation.

### 2.5. Cell Cytotoxicity and Proliferation

Cellular cytotoxicity and proliferation were measured using the Cell Counting Kit-8 reagent (CCK-8, Domino, Japan). Specifically, P34HB and P34HB-PPy scaffold membranes were cut to an appropriate size and placed in the bottom of a 96-well plate. The scaffold membranes were soaked in a mixed solution of absolute ethanol and PBS (v/v = 75%) for 12 h for sterilization and then washed with PBS 5 times to remove ethanol. Subsequently, the BMSCs were seeded into the P34HB and P34HB-PPy scaffold membranes at a cell density of 2 × 10^4^/well. The cells were placed in a carbon dioxide cell incubator (37°C, 5% CO_2_) and cultured until the cells returned to the normal adherent state. A 10% CCK-8 reagent was added to the corresponding wells at different time points (days 1, 3, and 5) and incubated for 2 h. Then, the obtained supernatant was transferred to another 96-well plate. The absorbance value of the solution was measured at 450 nm by a microplate reader (Varioskan, Thermo, USA), and the percentage of cell proliferation and viability was calculated via the following formula [43]:

(1) $$\begin{array}{c} \text{Relative cell viability}\left(\%\right)=\left[\left(\text{OD sample}-\text{OD medium blank}\right)/\left(\text{OD control}-\text{OD medium blank}\right)\right]\times 100\%. \end{array}$$

### 2.6. Fluorescence Staining

The effects of P34HB and P34HB-PPy fiber scaffolds on cell viability were observed by fluorescence staining. Briefly, BMSCs were seeded onto the scaffold membranes and cultured for 3 days. Then, all cell substrates were fixed on ice with 4% paraformaldehyde for 15 min followed by the permeabilization of the cells in 0.1% Triton-PBS for another 10 min. After each step, the cells were washed thrice with PBS. Subsequently, Actin Tracker Green (Beyotime, China) was used to visualize the cytoskeleton, whereas 4′6-diamidino-2-phenylindole (DAPI) (Beyotime, China) was used to visualize the cell nucleus. After staining in the dark, fluorescence images were acquired using a fluorescence microscope (Olympus IX73, Japan).

### 2.7. Assessment of the Cellular Morphology

The cellular morphology of the P34HB and P34HB-PPy scaffold membranes was assessed by SEM. The above cell substrates were fixed with a 3.0% glutaraldehyde aqueous solution for 15 min, washed thrice with PBS to remove residual glutaraldehyde, frozen overnight, and dehydrated using a freeze dryer for 48 h. Then, the cell substrates were sputter-coated with platinum/palladium at a thickness of 10 nm, and SEM images were obtained using a Hitachi model S-4800 system.

### 2.8. Statistical Analysis

Statistical analyses were performed using the SPSS software (version 22.0, USA). The results are expressed as the means ± standard deviations (SD). Statistical differences were determined using the one-way analysis of variance (ANOVA) and Student's *t*-test methods. A value of *p* < 0.05 was considered statistically significant.

## 3. Results and Discussion

### 3.1. Fabrication of the P34HB-PPy Scaffolds

**Figure 1:**
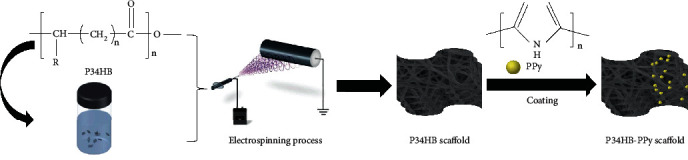
Fabrication of the core-shell structure of the P34HB-PPy composite fiber scaffold.

**Figure 2:**
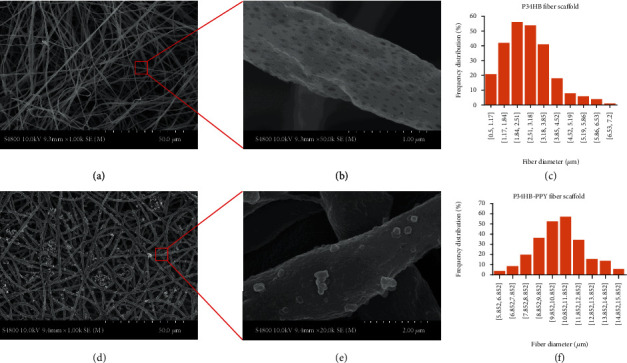
SEM images of the P34HB fiber scaffold (a, b) and P34HB-PPy composite fiber scaffold (d, e). The average diameter and size distribution of fibers in the P34HB scaffold (c) and P34HB-PPy composite scaffold (f).

In this paper, considering that P34HB needs to be dissolved in an appropriate organic solvent, the P34HB fiber scaffold was successfully manufactured using solution-assisted electrospinning technology (Figure 1). Specifically, the polymer powder was dissolved in DCM/DMF mixed organic solvent, and the electrostatic spinning technology was used to spray a trickle into the aqueous ethanol solution. The organic solvent rapidly diffuses in the aqueous ethanol solution. Then, the polymer solidifies into nanofibers. This wet electrostatic spinning technology can be used to remove harmful solvents quickly and easily to achieve green environmental protection. In addition, as shown in Figures 2(a) and 2(b) of SEM, all fibers were uniformly distributed, smooth and uniform in thickness without condensation, rupture, or collapse, and interconnected micropores were randomly arranged on the surface. The uniform pore structure may be related to the rapid diffusion of organic solvents. These structures promote soft and smooth scaffolds with better cell migration on the fiber structure. To further increase the hydrophilicity and cytocompatibility of the scaffold, the P34HB scaffold was modified by in situ electrochemical polymerization. Briefly, under the action of an electric field and oxidant, pyrrole monomer molecules will lose electrons on the surface of the P34HB fiber scaffold and become cationic free radicals. Then, the free radicals will combine with another monomer to become a dimer of pyrrole. After the chain growth step, the PPy macromolecular chain is finally obtained, which appears as a layer of PPy film covering the surface of the P34HB fiber scaffold. Figures 2(d) and 2(e) show that many unevenly distributed granular substances appeared on the surface of the smooth P34HB fiber scaffold, and the partially enlarged view shows that the microporous structure on the surface of the scaffold disappeared and was replaced by thin films and granular substances, which may be covered by the in situ polymerized PPy macromolecular chain. In addition, the appearance of the scaffolds changed from a smooth white film to a rough black film with a rougher outer layer structure. Therefore, we hypothesize that PPy was successfully coated on the surface of the scaffold, producing a core-shell structure composite scaffold of P34HB-PPy. Then, as shown in Figures 2(c) and 2(f), the fiber diameter distribution of the P34HB scaffold measured by the ImageJ software is between 0.5 *μ*m and 6.6 *μ*m with an average diameter of 2.7 *μ*m and fiber distance (aperture) of approximately 10-80 *μ*m. After coating with PPy, the diameter of the fiber is 5.85-15.62 *μ*m with an average diameter of 10.9 *μ*m. Therefore, the thickness of the PPy coating is approximately 5-10 *μ*m. After PPy in situ surface polymerization, the morphological distribution of the P34HB-PPY composite fibers is more uniform, and the fiber diameter is concentrated at approximately 11 *μ*m, indicating that the PPy coating will not significantly affect the fiber morphology of the scaffolds. Several studies have reported the effect of fiber size on cell adhesion [44, 45]. When the scale of cells is close to that of the fibers, the cells readily adhere to the thin fibers and maintain a high proliferation rate. For thick fibers, it is difficult for cells to adhere and proliferate. Therefore, our composite scaffold provides a more favorable microenvironment for cells, which has broad application prospects in cell attachment and growth.

### 3.2. Properties of the P34HB-PPy Scaffolds

**Figure 3:**
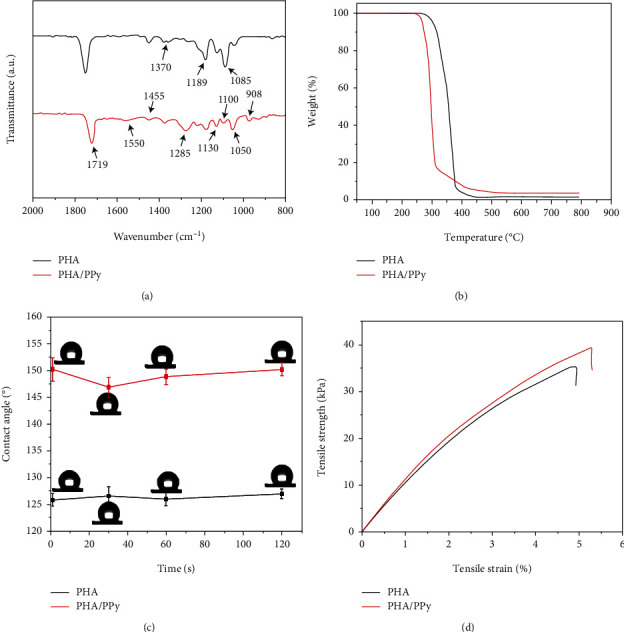
(a) Fourier transform infrared spectroscopy (FTIR) of the P34HB and P34HB-PPy scaffolds. (b) Thermogravimetric analysis of the P34HB and P34HB-PPy scaffolds. (c) The water contact angle of the P34HB and P34HB-PPy scaffolds. (d) The tensile strength curves of the P34HB and P34HB-PPy scaffolds.

The functional groups of P34HB and PPy polymers were determined by comparing the principal vibrational modes of the FTIR spectrum in Figure 3(a). The analysis of the spectra revealed that the major intense peaks recorded at 1719 cm^−1^ and 1285 cm^−1^ correspond to ester C=O bonds. The characteristic peaks of PPy at wavenumbers 1550 and 1455 cm^−1^ appeared in the FTIR spectra due to the symmetric and antisymmetric ring stretching modes, respectively [46]. Bands at 1189 and 908 cm^−1^ were assigned to the stretching vibration of doped PPy. In addition, bands at 1130, 1050, and 1370 cm^−1^ were attributed to the C–H deformation vibrations and the C–N stretching vibrations [47]. The peaks at 1100 and 1085 cm^−1^ were assigned to the C–O stretching vibration. The positions of the prominent bands matched those reported previously for PPy, which indicated the successful synthesis of PPy on the surface of the P34HB fiber scaffold.  Figure 3(b) shows the thermal stability of the sample. This technique is commonly used in the characterization of polymers because it can provide useful information about the intrinsic structure of the system. Single-phase thermal decomposition was shown in the TGA curve, and the weight loss was attributed to the thermal degradation of the P34HB fiber scaffold, where the initial weight loss began at 280°C. The mass change was observed between 300 and 400°C as the through curve affected substantial weight loss at the following temperatures: 300°C (97.14%), 350°C (56.72%), and 380°C (6.92%). In contrast, the thermal stability of the P34HB-PPy composite fiber scaffold is reduced, and the initial weight loss begins at 260°C, which is 20°C earlier than that of the P34HB scaffold. In other words, after treatment with PPy, the T max value decreases, which can be attributed to the decrease in polymorph II and the increase in amorphous cellulose [48]. The surface hydrophilicity of the material plays a key role in tissue engineering applications [49]. As shown in Figure 3(c), the surface of the pure P34HB fiber scaffold appeared hydrophobic, and the water contact angle was approximately 150.02 ± 1.64°. To increase the hydrophilicity of the material, we modified the PPy coating on the surface of the scaffold. The water contact angle was reduced to 125.05 ± 2.34°, and the hydrophilicity of the composite scaffold was greatly improved, which could greatly promote cell adhesion and proliferation. In addition, the tensile strength of the pure P34HB fiber scaffold was 34.9 kPa, and the elongation at break was 4.9%. The mechanical properties of the scaffold modified by PPy were improved. The corresponding tensile strength was 39.5 kPa, and the elongation at break was 5.4% (Figure 3(d)). In general, the thickness of the fiber membrane obtained by electrospinning technology gradually increases as the preparation time increases. Correspondingly, the mechanical properties of this fiber scaffold are positively correlated with the thickness. In this study, we prepared a layer of soft ultrathin P34HB-PPy fiber membrane, so it is foreseeable that its overall mechanical properties will not be strong. The reason for the production of a single-layer structure is its biomedical application considerations. When applied to the repair of human skin wounds, peritoneum and meninges, the soft structure can reduce the body's foreign body sensation, and the appropriate mechanical strength can support stable cell adhesion and growth. Studies have reported that combining collagen fiber scaffolds with ascorbic acid enhances collagen production and fibril cross-linking so that the tensile strength is significantly increased to approximately 57.8 kPa; thus, an engineered artificial blood vessel has been successfully constructed [50]. Our fiber scaffold is consistent with its mechanical properties, and we tested the proliferation and adhesion of human-derived bone marrow mesenchymal stem cells on the P34HB-PPy fiber scaffold, which can grow well under the existing tensile strength to meet the needs of tissue engineering. In the future, according to different application needs, such as cartilage repair and antitumor drug carriers, the preparation time or process of P34HB-PPy fiber scaffolds can be changed to obtain electrospun fiber scaffolds with excellent mechanical properties.

**Figure 4:**
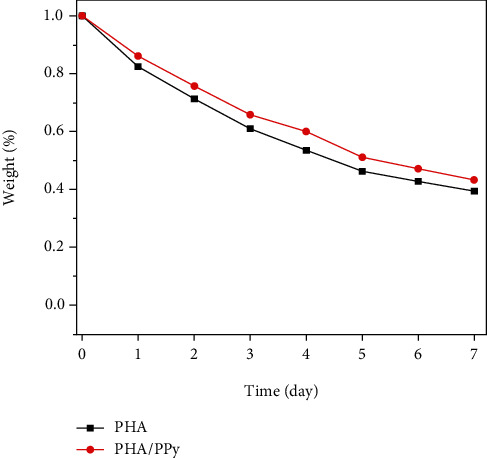
In vitro degradation test of P34HB and P34HB-PPy fiber scaffolds.

The degradability of the samples was evaluated by in vitro degradation experiments.  Figure 4 shows the enzymatic hydrolysis test of the weight loss of P34HB and P34HB-PPy composite fiber scaffolds with exposure time. The values of weight loss for both P34HB and P34HB-PPy scaffolds increased with prolonged exposure time and showed almost linear changes over 7 days, which allowed the enzymatic hydrolysis rates of the samples to be extracted from the slope of the curve. On the 7th day, the degradation rate of the P34HB and P34HB-PPy scaffolds stabilized, and the weight remained at approximately 40% of the initial weight, which may be affected by the polymer degradation method. Generally, there are two main routes of polymer degradation, namely, surface erosion and bulk erosion, and the degradation rates of different routes are obviously different [51]. In the early stage of this experiment, the sample showed bulk erosion in a high-concentration lipase (40 U) solution. The quality decreased rapidly, and then, it fragmented into small pieces. At this point, the degradation rate tends to be constant, and there is no obvious morphological change, indicating that the subsequent degradation may be facilitated by surface erosion. In addition, the faster degradation of P34HB may be due to the microporous structure on the surface. The increasing number of micropores may lead to an increase in the interface area between the degradation medium and P34HB scaffold under the same fiber diameter or volume, allowing the enzyme to gain access to a large number of P34HB chains, thus increasing the overall degradation rate. The P34HB-PPy composite scaffold is affected by the PPy core-shell structure, which can reduce the interface area between the sample and the degradation medium, thereby slowing the degradation rate. Based on these characteristics of the P34HB fiber scaffold, we can achieve controllable degradation and mechanical properties and customize fiber scaffolds with special properties in biomedical fields, such as skin wound repair, meningeal defect repair, and peritoneal defect repair, to take full advantage of individualized repair of tissue engineering fiber scaffolds.

### 3.3. In Vitro Cellular Assays

**Figure 5:**
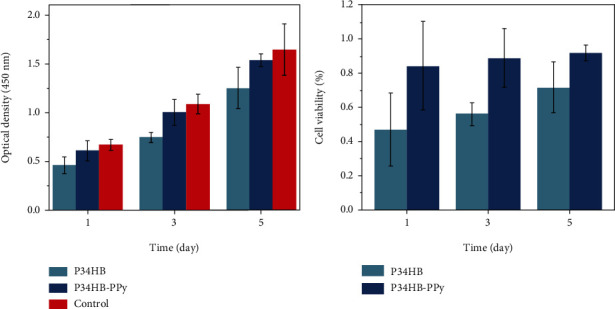
Cytotoxicity and proliferation of BMSCs cultured on P34HB and P34HB-PPy fiber scaffolds at various incubation periods.

CCK-8 was used to test the effects of P34HB and P34HB-PPy fiber scaffolds on cell culture. In this assay, the electron coupling reagent can be reduced by some dehydrogenases in the mitochondria of cells to form orange-yellow formazan, and the amount of formazan produced is proportional to the number of living cells. As the cell proliferates more rapidly, the color of the culture medium becomes darker. Measuring the absorbance value at 450 nm in a microplate reader can reflect the growth of cells. BMSCs were plated on fiber scaffolds that were cocultured for 5 days.  Figure 5 shows the absorbance value and cell viability on the P34HB and P34HB-PPy fiber scaffolds compared to the control sample on days 1, 3, and 5. The results showed that the absorbance values of the BMSCs at 450 nm on a P34HB fiber scaffold was 0.460, 0.745, and 1.252 on days 1, 3, and 5, respectively, and the calculated proliferation activity was 47.1%, 56.2%, and 71.8%, respectively. Correspondingly, the absorbance values at 450 nm on the P34HB-PPy composite scaffold were 0.609, 1.002, and 1.536, respectively, and the cell proliferation activity was 84.5%, 89.0%, and 92.1%, respectively. The absorbance values of the control group were 0.67, 1.088, and 1.647, respectively. The SPSS software analyses revealed that the differences between these groups were statistically significant (*p* = 0.013). The P34HB-PPy composite scaffold is more conducive to cell adhesion and proliferation, and cells can grow better on the nanofiber structure compared with the pure P34HB fiber scaffold. It has been reported that cell adhesion on the surface of the material is achieved through the biometric recognition process; the morphology and hydrophobicity of the surface will affect the adhesion and proliferation of cells [52, 53]. When the material is in contact with the cell culture medium, the surface of the material will quickly adsorb a layer of protein molecules. Then, the cells will contact the surface of the material. Thus, the cells adhere to the surface of the material through integrins, which subsequently proliferate. In this study, the addition of the PPy coating improved the hydrophobic properties of P34HB and increased the hydrophilicity and specific surface area of the scaffold. In addition, the charge properties and charge density of the material surface also have an important impact on cell growth. The electrostatic interaction between positively charged PPy and negatively charged cells is conducive to cell adhesion and rapid growth of biofilms, thereby providing an ideal microenvironment for the attachment and growth of cells.

**Figure 6:**
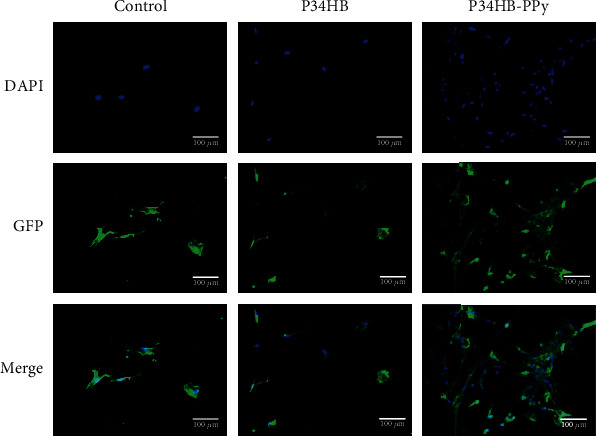
Fluorescence images of BMSCs cultured on the P34HB and P34HB-PPy fiber scaffolds on day 3. The scale bar was 100 *μ*m.

To further study the effect of P34HB and P34HB-PPy fiber scaffolds on cell proliferation, immunofluorescence staining of cultured BMSCs on the scaffolds was performed using DAPI and Actin Tracker Green to show cell morphology.  Figure 6 shows fluorescence images of the cytoskeleton (green) and cell nuclei (blue). In the control group and the P34HB scaffold, only a small number of BMSC cells were scattered. The density was very low. Only sparse intercellular contact was maintained, and the shape was slightly round. Further fluorescence quantitative analysis showed that the average fluorescence intensity of the P34HB group was 6.293 arbitrary units (AU), whereas the average fluorescence intensity of the P34HB-PPy group was 51.221 AU. This result is because the bottom surface of the control group is very smooth, whereas P34HB is relatively hydrophobic and hinders cell attachment, resulting in poor cell retention ability, which is not conducive to cell growth and proliferation. The BMSCs fell off during a series of staining and rinsing steps in the experiment, yielding the noted cell morphology, as shown in Figure 5. In sharp contrast, the BMSCs displayed normal cytoskeleton and cell nuclei morphology on the P34HB-PPy composite scaffolds. The cells were distributed in a spindle-like shape and formed a higher-density homogeneous cell layer, exhibiting better cell attachment, cell spreading, and cell retention performance. This finding was consistent with the above CCK-8 cell proliferation results.

**Figure 7:**
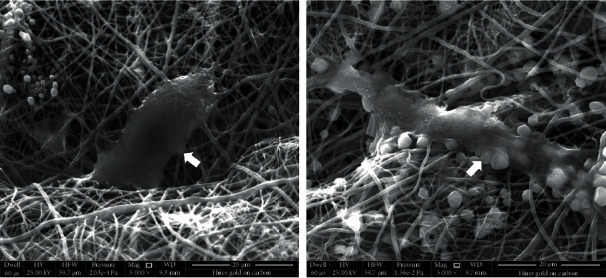
SEM images of BMSCs cultured on the P34HB and P34HB-PPy fiber scaffolds on day 3.

After the BMSCs were cocultured with the scaffold for 3 days, the detailed cell morphology on the scaffold was observed through SEM. As shown by the arrows in Figure 7, the cells on the pure P34HB fiber scaffold were scattered in small numbers. The overall density was low, and the cells were loosely connected with each other. In contrast, the BMSCs on P34HB-PPy composite scaffolds showed extensive intercellular contact and a tight connection with the scaffolds. The cells grew much better and even fused with each other to form clusters from the free migration of cells on the scaffolds, making it difficult to distinguish the morphology of individual BMSCs. This behavior helps maintain cell viability and function and further promotes cell proliferation.

These results indicate that the P34HB-PPy composite scaffold not only provides a sufficient nanofiber microenvironment for cell attachment and proliferation but also provides durable support with stable mechanical strength. Interestingly, the growth and proliferation of cells on the composite scaffold is not only due to PPy improving the hydrophilicity of the P34HB surface but also mostly because it increases the cell retention capacity, thus exerting the active promotion effect of the P34HB material and its degradation products on cells. Previous studies have reported that PHA substances can significantly promote cell growth and proliferation [33, 54] probably due to its degradation product, 3-hydroxybutyric acid (3-HB), one of the ketone substances produced by the oxidative decomposition of fatty acids. This substance is an important source of capacity for brain and muscle tissues and can replace glucose for an energy supply during starvation or an insufficient sugar supply [55]. Therefore, PHA substances can provide basic nutrients for cells during cultivation to promote cell and tissue regeneration. These fiber scaffolds have shown promising applications in the field of tissue regeneration. Nanofiber core-shell scaffolds with aligned structures are used for peripheral nerve tissue regeneration [56], and anisotropic cardiac structure scaffolds are used for cardiac tissue regeneration [57]. These 3D hybrid scaffolds prepared by electrospinning technology can induce cellular orientation, maturation, and anisotropy and ultimately achieve precise repair of the defect area. In addition, nanofiber wound dressings with multifunctional properties that integrate suitable mechanical properties, electroactivity, antioxidants, and inherent antibacterial activity are also one of the research hotspots of fiber scaffolds. This is the most likely direction for product transformation in the field of bionics [58].

## 4. Conclusions

In conclusion, we successfully prepared a novel core-shell fiber scaffold through simple and versatile electrospinning and in situ surface polymerization technology. The obtained P34HB-PPy composite scaffold has a rich spatial network structure and shows good mechanical properties and cell compatibility through a universal material testing machine, contact angle measuring system, thermogravimetric analyzer, and other methods. The results showed that the hydrophilicity of the P34HB-PPy composite scaffold was significantly improved, and the simulated ECM microenvironment had excellent cell retention and promotion ability. This scaffold can form a complete fiber construct with cells. More importantly, P34HB is a fully degradable material, and its degradation product 3HB is one of the body's energy metabolites that can promote cell growth and proliferation. Therefore, we expect that the P34HB-PPy composite scaffold can be widely used in biomedical fields, such as tissue engineering and soft tissue repair.

## Data Availability

Data can be available upon request to the corresponding author.
